# Guanidinoacetate Is More Effective than Creatine at Enhancing Tissue Creatine Stores while Consequently Limiting Methionine Availability in Yucatan Miniature Pigs

**DOI:** 10.1371/journal.pone.0131563

**Published:** 2015-06-25

**Authors:** Laura E. McBreairty, Jason L. Robinson, Kayla R. Furlong, Janet A. Brunton, Robert F. Bertolo

**Affiliations:** Department of Biochemistry, Memorial University of Newfoundland, St. John’s, Newfoundland and Labrador, Canada; Mayo Clinic, UNITED STATES

## Abstract

Creatine (Cr) is an important high-energy phosphate buffer in tissues with a high energy demand such as muscle and brain and is consequently a highly consumed nutritional supplement. Creatine is synthesized via the S-adenosylmethionine (SAM) dependent methylation of guanidinoacetate (GAA) which is not regulated by a feedback mechanism. The first objective of this study was to determine the effectiveness of GAA at increasing tissue Cr stores. Because SAM is required for other methylation reactions, we also wanted to determine whether an increased creatine synthesis would lead to a lower availability of methyl groups for other methylated products. Three month-old pigs (*n* = 18) were fed control, GAA- or Cr-supplemented diets twice daily. On day 18 or 19, anesthesia was induced 1–3 hours post feeding and a bolus of [methyl-^3^H]methionine was intravenously infused. After 30 minutes, the liver was analyzed for methyl-^3^H incorporation into protein, Cr, phosphatidylcholine (PC) and DNA. Although both Cr and GAA led to higher hepatic Cr concentration, only supplementation with GAA led to higher levels of muscle Cr (*P* < 0.05). Only GAA supplementation resulted in lower methyl-^3^H incorporation into PC and protein as well as lower hepatic SAM concentration compared to the controls, suggesting that Cr synthesis resulted in a limited methyl supply for PC and protein synthesis (*P* < 0.05). Although GAA is more effective than Cr at supporting muscle Cr accretion, further research should be conducted into the long term consequences of a limited methyl supply and its effects on protein and PC homeostasis.

## Introduction

In the cell, creatine (Cr) functions as a high-energy phosphate buffer via its conversion to phosphocreatine and subsequent restoration of ATP by the enzyme creatine kinase. In an omnivorous adult, both diet and endogenous synthesis play an equal role in replenishing the daily Cr loss [1] of approximately 1.7% per day [2]. Endogenous Cr synthesis is primarily a dual organ process originating in the kidney with the synthesis of guanidinoacetate (GAA) from arginine and glycine via the enzyme arginine:glycine amidinotransferase (AGAT). GAA is taken up primarily by the liver and converted to Cr by guanidinoacetate N-methyltransferase (GAMT) which transfers a methyl group to GAA from S-adenosylmethionine (SAM), a universal methyl donor synthesized from methionine [3]. This reaction also produces homocysteine, a known risk factor for cardiovascular disease [4].

Creatine has been extensively studied as a nutritional supplement to improve exercise and sports performance [5–7] and has recently gained attention as a potential neuroprotectant and therapeutic agent for neurodegenerative disease [8–10]. The agriculture industry has also been interested in Cr as a feed supplement due to its role in energy metabolism and its demonstrated ability to spare arginine [11], which cannot be directly supplemented due to antagonistic effects on other amino acids [12]. More recently, GAA’s favorable cost and stability attributes have led to consideration of GAA as an alternative to Cr supplementation [13]. In chicks fed an arginine-deficient diet, both GAA and Cr supplementation resulted in a higher gain:feed ratio [14]. Moreover, GAA supplementation to a Cr-deficient diet in chicks effectively increased levels of muscle Cr and breast muscle yield [15]. Human studies have also demonstrated a dose response effect of GAA supplementation on plasma levels of both GAA and Cr [16]. Although the beneficial effects of Cr supplementation have been extensively studied, less is known about the effectiveness of GAA supplementation as a means to increase Cr stores. Moreover, the capacities of GAA and Cr to increase levels of tissue Cr have not been compared. The first objective of this study was to determine whether GAA or Cr was more effective at enhancing the tissue stores of Cr in pigs.

However, the possible metabolic consequences of GAA supplementation must also be examined. Hepatic synthesis of Cr utilizes a significant proportion of methyl groups [17] which could potentially limit methyl availability for other important transmethylation products such as phosphatidylcholine (PC) and methylated DNA. Furthermore, because the methyl group donated by SAM originates from methionine, increasing methyl group demand for Cr synthesis could potentially limit methionine availability for protein synthesis. Indeed, we have recently shown in suckling piglets that an acute 2-hour intraportal infusion of GAA doubled methyl incorporation into Cr and simultaneously reduced methyl incorporation into PC by 80% and methyl incorporation into protein by 40% [18]: however, it is not known whether chronic dietary supplementation with GAA leads to a similar reduction in methyl availability or whether these effects lead to changes in growth and levels of hepatic PC. Conversely, supplemental Cr has the potential to spare methyl groups via negative feedback on AGAT activity [19], lowering GAA production and the demand for methyl groups used in Cr synthesis, as well as lowering homocysteine production, which has been demonstrated in Cr supplemented rats [20]. The second objective of this study was to elucidate the mechanistic effects of dietary GAA and Cr supplementation on the partitioning of methionine among transmethylation products and protein synthesis.

## Materials and Methods

### Reagents

L-[Methyl-^3^H]methionine was obtained from American Radiolabeled Chemicals, Inc. (St. Louis, MO). All other chemicals were of analytical grade and were from Sigma (St. Louis, MO) or Fisher Scientific (Fair Lawn, NJ).

### Animals and experimental design

All animal handling procedures (protocol: 11-55-RB) were performed at Memorial University and approved by the Institutional Animal Care Committee in accordance with the guidelines of the Canadian Council on Animal Care. Isoflurane anesthesia was used as described below and all efforts were made to minimize suffering. Eighteen pre-pubertal Yucatan miniature pigs (14–16 weeks old) were obtained from Memorial University of Newfoundland breeding colony and weight-matched to control (n = 6), GAA supplemented (n = 6) or Cr supplemented (n = 6) groups. Pigs were group housed in a large room with ample space to run and were supplied with rubber balls for enrichment. Pigs had unlimited access to water and received standard pig grower diet which was fed twice daily. Supplemental GAA (157 mg/kg/day) or Cr monohydrate (200 mg/kg/day) was mixed with an aliquot of grower feed and hand-fed to experimental groups during each feeding for 18–19 days. The supplementation level in this study roughly corresponds to a typical Cr dosing schedule (i.e., 20 g/day loading, 2–5 g/day maintenance) used by humans to increase muscle Cr levels and achieve positive ergogenic effects [21,22]. On the final day of supplementation, pigs received their morning feeding and ~1 hour later, anesthesia was induced with isoflurane (1–2%) in oxygen (1.5 L/minute) to reduce suffering. Two catheters were inserted into the right and left jugular veins and pigs received an intravenous bolus infusion of 0.75 mCi of L-[methyl-^3^H]methionine via one catheter while the other was used to sample blood before and every 7 min after the bolus infusion. Thirty min following the infusion, a laparotomy was performed using cautery and the liver, right kidney, heart, brain and sample of biceps femoris muscle were immediately excised and freeze clamped. Pigs were euthanized via exsanguination while under anesthesia. A labeling period of 30 minutes following the bolus of L-[methyl-^3^H]methionine was chosen based on a previous study [18] demonstrating that incorporation of label into DNA and PC was linear from 30–60 minutes while incorporation into Cr was constant during that time. Measurements were made during the fed state in a controlled manner. Others have shown that following a bolus meal, hepatic protein synthesis plateaus at 30 minutes and returns to baseline levels by 4 hours in piglets [23]; in our study, all tissues were collected within 2–3 hours following feeding.

### Plasma and liver metabolite concentrations

Total homocysteine, cysteine and glutathione concentrations were determined by HPLC according to [24]. Plasma and tissue Cr and GAA concentrations were determined by HPLC following derivatization with ninhydrin [25]. Liver SAM and S-adenosyl-L-homocysteine (SAH) concentrations were determined by HPLC [26]. Plasma and liver amino acid concentrations were determined by HPLC using phenylisothiocyanate derivatization [27]. Plasma samples were first deproteinized with 0.5% trifluoroacetic acid in methanol. Liver samples were homogenized in perchloric acid; the supernatant was used to determine free amino acids and the protein pellet was hydrolyzed in 6 M HCl for 24 h and used to determine tissue-bound amino acids. Specific radioactivity (SRA) of SAM (DPM/μmol) was determined by fraction collecting the peak and DPM were determined by scintillation counting (Perkin Elmer Canada Ltd, Woodbridge, ON, Canada).

### Hepatic analyses of methylated products

Creatine SRA and total Cr concentration were determined using a modified method [18] by Lamarre et al. [28]. For PC analyses, lipids were extracted from liver using the method by Folch et al. [29] and separated via thin layer chromatography as previously described [18]. Total phosphorus was determined using a modified Bartlett method [30] and PC SRA determined by scintillation counting [18]. DNA was extracted and SRA (DPM/μg) determined as previously described [18].

### Calculations

Rate of ^3^H-methyl incorporation into methylated products and liver protein in 30 min was calculated as:

$${\text{Rate of}}^{3}{\text{H-methyl incorporation}}_{\text{product}}=\left({\text{SRA}}_{\text{product}}/{\text{SRA}}_{\text{precursor}}\right)\times 100,$$

where the intracellular precursor was hepatic SAM for all products. We found that in some analyses, tissue free methionine SRA is highly variable due to mixing with blood free methionine in the sample. Because tissue free SAM SRA is well equilibrated with intracellular methionine SRA, SAM was used as a representative intracellular precursor for protein synthesis, SAM was used as a representative precursor for protein synthesis as well as for methylated products.

### Statistical analyses

Data are presented as means ± SD. All normally distributed data were analyzed using one-way ANOVA with Newman-Keuls multiple comparison post-tests when main effects were identified. GAA concentrations were not normally distributed as determined by the D’Agostino Pearson normality test, so non-parametric Kruskal-Wallis analysis was used to determine differences among groups with Dunn’s post-tests when main effects were identified (GraphPad Prism 4.0 for Windows, GraphPad Software, San Diego, CA). Differences were considered significant at *P* < 0.05.

## Results

### Body weight

The percent body weight change from day 0 to day 18–19 of supplementation was 23% ± 6, 22% ± 4 and 28% ± 5 for control, Cr supplemented and GAA supplemented pigs, respectively, with no difference among groups.

### Tissue and plasma creatine and GAA

Compared to control pigs, GAA and Cr supplementation led to ~7.5- and ~4-fold higher hepatic Cr concentrations, respectively, and hepatic Cr was ~2-fold higher with GAA versus Cr supplementation (Table 1). Muscle Cr was ~20% higher in GAA supplemented pigs compared to control pigs while Cr supplementation was intermediate with no difference from control or GAA. Both Cr and GAA supplementation led to a higher Cr concentration in the kidney compared to the control group while Cr concentrations in the heart and brain were not different among groups. GAA concentration was higher in the kidney following GAA supplementation compared to both other groups. GAA supplemented pigs had higer hepatic and muscle GAA concentrations compared to controls with no differences in heart or brain GAA concentrations among groups (Table 2). Plasma Cr concentrations were 70% higher than control with GAA supplementation and 140% higher with Cr supplementation; moreover, Cr supplementation led to 40% higher Cr concentrations compared to GAA supplementation (Table 3). Plasma GAA concentrations were higher with GAA supplementation compared to both Cr and control groups (Table 3).

**Table 1:** Tissue distribution of creatine in pigs fed a control, creatine supplemented or GAA supplemented diet for 18–19 days^2^.

	Control	Creatine	GAA^1^
	Creatine, μmol/g wet weight		
Liver	0.429 ± 0.14^a^	1.674 ± 0.61^b^	3.125 ± 1.32^c^
Muscle	39.90 ± 2.42^a^	44.08 ± 4.10^ab^	47.63 ± 4.25^b^
Kidney	0.418 ± 0.08^a^	1.132 ± 0.38^b^	0.853 ± 0.19^b^
Heart	19.22 ± 4.49	18.07 ± 2.87	19.34 ± 3.99
Brain	6.07 ± 1.40	5.51 ± 1.55	7.49 ± 3.92

^1^ GAA, guanidinoacetate

^2^Data are means ± SD; *n* = 6. Means with different superscripts are significantly different within rows, *P* < 0.05.

**Table 2:** Tissue distribution of GAA^1^ in pigs fed a control, creatine supplemented or GAA supplemented diet for 18–19 days^2^.

	Control	Creatine	GAA
Liver	7.38 ± 2.7^a^	16.8 ± 10^ab^	1323 ± 1796^b^
Muscle	44.2 ± 14^a^	73.7 ± 43^ab^	92.0 ± 36^b^
Kidney	81.2 ± 23^a^	97.2 ± 29^a^	230 ± 83^b^
Heart	60.5 ± 41	73.6 ± 32	93.4 ± 20
Brain	24.0 ± 7.0	20.8 ± 3.7	28.1 ± 12.3

GAA, nmol/g wet weight

^1^ GAA, guanidinoacetate

^2^Data are means ± SD; *n* = 6. Means with different superscripts are significantly different within rows, *P* < 0.05.

**Table 3:** Plasma concentrations of metabolites in pigs fed a control, creatine supplemented or GAA^1^ supplemented diet for 18–19 days^2^.

	Control	Creatine	GAA
	μmol/L		
Homocysteine	17.8 ± 1.5^a^	16.1 ± 1.7^a^	34.2 ± 13.3^b^
Cysteine	50.3 ± 7.4	48.5 ± 7.2	60.4 ± 9.5
Glutathione	43.6 ± 6.8	49.5 ± 8.7	37.1 ± 6.2
Creatine	112 ± 45^a^	264 ± 59^c^	186 ± 37^b^
GAA	4.3 ± 1.4^a^	4.1 ± 1.1^a^	49.0 ± 45.4^b^

^1^ GAA, guanidinoacetate

^2^Data are means ± SD; *n* = 6. Means with different superscripts are significantly different within rows, *P* < 0.05.

### Rate of ^3^H-methyl incorporation into transmethylation products

In GAA supplemented pigs, the rate of ^3^H-methyl incorporation into Cr was ~4-fold and ~3-fold higher than control and Cr supplemented pigs, respectively (Table 4). Compared to control and Cr supplemented groups, GAA supplementation also resulted in ~75% lower ^3^H-methyl incorporation into PC and ~50% lower ^3^H-methyl incorporation into hepatic protein, with no difference between Cr supplemented and control pigs. There were no differences in ^3^H-methyl incorporation into DNA or in the SRA of SAM among groups (Table 4).

**Table 4:** Rate of ^3^H-methyl incorporation into transmethylation products and hepatic protein after 30 minutes (corrected for SAM) in pigs fed a control, creatine supplemented or GAA supplemented diet for 18–19 days^1^.

	Control	Creatine	GAA
	Rate of ^3^H-methyl incorporation^2^		
Creatine	23.2 ± 7.0^a^	32.5 ± 12.6^a^	89.4 ± 45.3^b^
PC	4.24 ± 1.40^b^	3.34 ± 0.86^b^	0.95 ± 0.51^a^
DNA	248 ± 90	249 ± 71	193 ± 42
Protein	2.73 ± 0.87^b^	2.48 ± 0.69^b^	1.22 ± 0.42^a^

^1^Data are means ± SD; *n* = 6. Means with different superscripts are significantly different within rows, *P* < 0.05. GAA, guanidinoacetate; PC, phosphatidylcholine; SAM, S-adenosylmethionine; SRA, specific radioactivity.

^2^Corrected rate of ^3^H-methyl incorporation = (SRA_product_ / SRA_precursor_) × 100

### Plasma and liver metabolite concentrations

Supplementation with GAA resulted in a ~50% lower hepatic SAM concentration compared to both control and Cr supplemented groups while there were no differences in hepatic SAH or SAM/SAH ratio among groups (Table 5). There were also no differences in hepatic methionine, arginine or PC among groups. GAA supplemented pigs had ~2-fold higher plasma homocysteine compared to control and Cr supplemented pigs with no difference in plasma cysteine or glutathione among groups (Table 3).

**Table 5:** Hepatic concentrations of metabolites in pigs fed a control, creatine supplemented or GAA supplemented diet for 18–19 days^1^.

	Control	Creatine	GAA
	nmol/g wet weight		
Arginine	43.2 ± 4.6	64.3 ± 35.5	41.6 ± 4.1
Methionine	99.1 ± 38.4	99.3 ± 30.1	86.6 ± 28.5
SAM	56.9 ± 8.4^b^	66.5 ± 22.6^b^	31.9 ± 16.5^a^
SAH	15.1 ± 2.8	15.4 ± 2.6	18.3 ± 11.8
SAM/SAH	3.94 ± 1.24	4.35 ± 1.50	2.46 ± 1.68
SAM DPM	114 ± 28	127 ± 33	144 ± 47
PC (umol/g)	8.1 ± 2.2	8.5 ± 2.4	6.9 ± 2.9

^1^Data are means ± SDs; *n* = 6. Means with different superscripts are significantly different within rows, *P* < 0.05. DPM, disintegrations per minute; GAA, guanidinoacetate; PC, phosphatidylcholine; SAH, S-adenosylhomocysteine; SAM, S-adenosylmethionine

## Discussion

We supplemented young pigs with equimolar amounts of either GAA or Cr for 18–19 days and found that while both GAA and Cr supplementation led to higher Cr concentrations in the liver and kidney compared to control pigs, GAA supplementation was more effective than Cr at increasing hepatic Cr concentrations. Furthermore, only GAA supplemented pigs had a higher concentration of muscle Cr compared to control pigs, suggesting supplemental GAA could be a potential approach to increase body Cr stores.

Because of the positive ergogenic and therapeutic effects of Cr, the use of dietary Cr supplementation to increase muscle concentrations has been a growing area of interest. Although experiments in both humans and animals have demonstrated that dietary Cr supplementation can be an effective means to increase muscle Cr [22], some studies have yielded inconsistent findings [31]. Similar to our results, the relative increase in tissue Cr following supplementation is higher in tissues with low levels of basal Cr (i.e., kidney and liver) compared to tissues with high basal Cr concentrations (i.e., skeletal muscle, heart and brain) [31,32]. However, it is notable that our level of supplementation of either Cr or GAA did not enhance heart or brain concentrations of either metabolite. Skeletal muscle has a limited capacity to store Cr [22] and it has been proposed that individuals classified as “non-responders” to Cr supplementation have higher initial Cr concentrations [33]. Because of both the limited Cr storage capacity of skeletal muscle [22] and the high relative basal Cr concentration of the biceps femoris muscle [34] sampled in this study, it is possible that despite a ~2.5-fold increase in plasma Cr concentrations, the Cr concentration of the muscle sampled in this study was at a maximal Cr storage capacity prior to supplementation and did not take up circulating Cr. Indeed, others have shown that white gastrocnemius muscle had lower Cr uptake following supplementation when compared to a pre-supplementation rate, a finding that was not observed in other muscle types with lower basal Cr concentrations [35]. Because of the limited potential for supplemental Cr to augment muscle Cr stores, studies have investigated alternate methods to increase the muscle’s capacity for Cr uptake [36,37].

Although in this study supplemental Cr had no significant effect on muscle concentrations of the metabolite, it is interesting that GAA supplementation both increased Cr in the muscle and was more effective than Cr supplementation at increasing concentrations in the liver. Because the liver is the primary site for Cr synthesis, the higher level of hepatic Cr following GAA supplementation is a result of endogenous synthesis in the liver. However, the mechanism leading to a higher level of muscle Cr could be a result of transport of hepatic Cr, endogenous synthesis in the muscle or a combination of both. Higher concentrations of extracellular Cr have been shown to downregulate Cr transport into rat muscle, with 50% inhibition occuring at physiological Cr concentrations [38]. So it is possible that the higher plasma Cr levels found in our Cr supplemented pigs led to more downregulation of the Cr transporter, compared to GAA supplemented pigs. This downregulation could also explain contrasting findings in rats that showed Cr supplementation is more effective than GAA at enhancing total Cr concentration in muscle, probably because plasma Cr concentrations were unchanged in that study [17].

Although less is known about GAA transport, experiments using isolated rat hepatocytes demonstrate a higher GAA uptake activity compared to Cr uptake [39]. In rats, the K_m_ of the transporter for Cr is similar to plasma Cr concentrations. In contrast, under normal conditions, plasma GAA concentration is more than 90% lower than the K_m_ for gamma-aminobutyric acid transporter-2, a proposed transporter for hepatic GAA [39]; however, in our GAA supplemented pigs, plasma GAA concentrations were 10-fold higher, approximating the estimated K_m_ for its transporter, suggesting higher transport of GAA into muscle following supplementation. Although the liver has the highest level of GAMT activity, detectable levels have been found in the muscle of young pigs [40] so this transported GAA could be rapidly converted to Cr. GAA supplementation was more effective at enhancing levels of tissue Cr, even though both Cr and GAA supplementation resulted in high plasma Cr concentrations; thus, it seems likely that the mechanism responsible for increasing muscle Cr is both transport of Cr from the liver as well as endogenous synthesis in the muscle. These results suggest GAA supplementation is an effective means to raise muscle Cr concentration beyond the level obtainable from Cr supplementation alone.

We previously demonstrated in an acute model of GAA supplementation that Cr synthesis is not feedback regulated at GAMT [18], and others have produced the same effect in rats [41]. In this study, we investigated whether chronic supplementation of GAA would increase Cr synthesis thereby limiting methyl group availability for other methylation reactions. As expected, supplementation with GAA led to a higher rate of incorporation of methyl groups into Cr (Table 4). Concomitantly, chronic supplementation with GAA also resulted in reduced methyl incorporation into both PC and protein, with no change in incorporation into DNA. These results are in keeping with our previous findings of increased methyl incorporation into Cr and lower methyl incorporation into PC and protein following an acute intraportal infusion of GAA in piglets [18]. We hypothesized that chronic Cr supplementation would lead to lower methyl incorporation into creatine and spare methyl groups for other transmethylation reactions; however, in this study chronic Cr supplementation did not affect creatine or PC incorporation of methyl groups suggesting creatine did not spare methyl groups.

Interestingly, unlike rats, Cr supplementation did not lead to lower plasma homocysteine levels [20]. The kidney is the primary site for GAA synthesis in the rat while the kidney only accounts for 20% of GAA production in humans [42]. Although piglets have significant AGAT activity in the kidney, the AGAT activity in the pancreas is 5-fold higher, suggesting that the pancreas may be the primary site for GAA production [40] and it is possible that GAA production in the pig pancreas may not be regulated via feedback inhibition of AGAT, as supported by the similar plasma GAA concentrations in Cr supplemented and control pigs.

Although an omnivorous adult human typically synthesizes half of their Cr requirement [1], it is not known whether this is similar in a weaned pig. In this study, the pig grower diet provided 0.05 mmol/kg/day of Cr via meat meal [43], which is ~5% of the total Cr accrued by a growing piglet (i.e., ~0.8 mmol/kg/day) [40]. With minimal Cr provided in the diet, it is likely that the pigs in this study must have synthesized the vast majority of their Cr needs. Based on this assumption, the GAA concentration in our diet (1.53 mmol/kg/day) provided approximately twice the amount required to synthesize the daily Cr requirement. Doubling the methyl group requirement for Cr synthesis via GAA supplementation helps explain the limited methyl availability for other transmethylation reactions. This hypothesis is supported by the lower hepatic SAM concentration in GAA supplemented pigs which interestingly was not accompanied by a lower hepatic concentration of the transmethylation product SAH, a known inhibitor of transmethylation reactions [44]. Given the higher levels of plasma homocysteine with GAA supplementation, it is likely that SAH was readily converted to homocysteine and exported into the plasma.

The effectiveness of GAA at increasing both hepatic and muscle Cr concentrations suggests GAA may be more favorable than Cr at increasing Cr stores. However, the higher methyl group utilization for Cr synthesis following supplementation with GAA can lead to a limited methyl group supply for other transmethylation reactions. Although a 6 week GAA supplementation trial in humans concluded that GAA is safe with a low incidence of biochemical abnormalities and an acceptable side-effects profile [45], the potential for GAA supplementation to limit protein and PC synthesis in a growing animal is concerning and warrants consideration. Total hepatic PC concentration was not different in GAA supplemented pigs, likely becauase PEMT is thought to be responsible for only ~30% of hepatic PC synthesis, with the remainder synthesized by the enzyme CDP-choline:1,2-diacylglycerol cholinephosphotransferase [46]. It is possible that this alternate pathway for PC synthesis compensated for lower PC synthesis via PEMT; however, studies in PEMT knockout mice demonstrate that the PEMT pathway is specifically important for very low-density lipoprotein secretion [47]. Because we supplemented with GAA twice daily, it is possible that the limited methyl group availability is an acute post-prandial response to GAA so future studies should investigate long term outcomes with respect to methyl group incorporation and protein synthesis. In particular, the 40% lower incorporation into protein with GAA supplementation is likely an acute effect considering growth was not affected in these pigs, although body composition was not assessed. In spite of the effectiveness of GAA at enhancing Cr stores, more research is needed to clarify the potentially detrimental effects of GAA supplementation.

## References

[1] Stead LM, Brosnan JT, Brosnan ME, Vance DE, Jacobs RL. Is it time to reevaluate methyl balance in humans? Am J Clin Nutr. 2006;83: 5–10. 16400042 10.1093/ajcn/83.1.5

[2] Wyss M, Kaddurah-Daouk R. Creatine and creatinine metabolism. Physiol Rev. 2000;80: 1107–1213. 10893433 10.1152/physrev.2000.80.3.1107

[3] Walker JB. Creatine: biosynthesis, regulation, and function. Adv Enzymol Relat Areas Mol Biol. 1979;50: 177–242. 386719 10.1002/9780470122952.ch4

[4] Ueland PM, Refsum H, Beresford SA, Vollset SE. The controversy over homocysteine and cardiovascular risk. Am J Clin Nutr. 2000;72: 324–332 10919921 10.1093/ajcn/72.2.324

[5] Bemben MG, Lamont HS. Creatine supplementation and exercise performance: recent findings. Sports Med. 2005;35: 107–125. 15707376 10.2165/00007256-200535020-00002

[6] Barber JJ, McDermott AY, McGaughey KJ, Olmstead JD, Hagobian TA. Effects of combined creatine and sodium bicarbonate supplementation on repeated sprint performance in trained men. J Strength Cond Res. 2013;27: 252–258. 10.1519/JSC.0b013e318252f6b7 23254493

[7] Dabidi Roshan V, Babaei H, Hosseinzadeh M, Arendt-Nielsen L. The effect of creatine supplementation on muscle fatigue and physiological indices following intermittent swimming bouts. J Sports Med Phys Fitness. 2013;53: 232–239. 23715246

[8] Peña-Altamira E, Crochemore C, Virgili M, Contestabile A. Neurochemical correlates of differential neuroprotection by long-term dietary creatine supplementation. Brain Res. 2005;1058: 183–188. 16140286 10.1016/j.brainres.2005.07.011

[9] Bender A, Samtleben W, Elstner M, Klopstock T. Long-term creatine supplementation is safe in aged patients with Parkinson disease. Nutr Res. 2008;28: 172–178. 10.1016/j.nutres.2008.01.001 19083405

[10] Hersch SM, Gevorkian S, Marder K, Moskowitz C, Feigin A, Cox M, et al Creatine in Huntington disease is safe, tolerable, bioavailable in brain and reduces serum 8OH2'dG. Neurology. 2006;66: 250–252. 16434666 10.1212/01.wnl.0000194318.74946.b6

[11] Austic RE, Nesheim MC. Arginine and creatine interrelationships in the chick. Poult Sci. 1972;51: 1098–1105. 4647572 10.3382/ps.0511098

[12] Chamruspollert M, Pesti GM, Bakalli RI. Dietary interrelationships among arginine, methionine, and lysine in young broiler chicks. Br J Nutr. 2002;88: 655–660. 12493087 10.1079/BJN2002732

[13] Baker DH. Advances in protein–amino acid nutrition of poultry. Amino Acids. 2009;37: 29–41. 10.1007/s00726-008-0198-3 19009229

[14] Dilger RN, Bryant-Angeloni K, Payne RL, Lemme A, Parsons CM. Dietary guanidino acetic acid is an efficacious replacement for arginine for young chicks. Poult Sci. 2013;92: 171–177. 10.3382/ps.2012-02425 23243244

[15] Michiels J, Maertens L, Buyse J, Lemme A, Rademacher M, Dierick NA, et al Supplementation of guanidinoacetic acid to broiler diets: Effects on performance, carcass characteristics, meat quality, and energy metabolism. Poult Sci. 2012;91: 402–412. 10.3382/ps.2011-01585 22252354

[16] Ostojic SM, Stojanovic M, Drid P, Hoffman JR. Dose-response effects of oral guanidinoacetic acid on serum creatine, homocysteine and B vitamins levels. Eur J Nutr. 2014;53: 1637–1643. 10.1007/s00394-014-0669-0 24535415

[17] Stead LM, Au KP, Jacobs RL, Brosnan ME, Brosnan JT. Methylation demand and homocysteine metabolism: effects of dietary provision of creatine and guanidinoacetate. Am J Physiol Endocrinol Metab. 2001;281: 1095–1100. 10.1152/ajpendo.2001.281.5.E1095 11595668

[18] McBreairty LE, McGowan RA, Brunton JA, Bertolo RF. Partitioning of [methyl-^3^H]methionine to methylated products and protein is altered during high methyl demand conditions in young Yucatan miniature pigs. J Nutr. 2013;143: 804–809. 10.3945/jn.112.172593 23616507

[19] McGuire DM, Gross MD, van Pilsum JF, Towle HC. Repression of rat kidney L-arginine:glycine amidinotransferase synthesis by creatine at a pretranslational level. J Biol Chem. 1984;259: 12034–12038. 6384218

[20] Deminice R, Portari GV, Vannucchi H, Jordao AA. Effects of creatine supplementation on homocysteine levels and lipid peroxidation in rats. Br J Nutr. 2009;102: 110–116. 10.1017/S0007114508162985 19079843

[21] Gordon A, Hultman E, Kaijser L, Kristjansson S, Rolf CJ, Nyquist O, et al Creatine supplementation in chronic heart failure increases skeletal muscle creatine phosphate and muscle performance. Cardiovasc Res. 1995;130: 413–418. 7585833

[22] Harris RC, Söderlund K, Hultman E. Elevation of creatine in resting and exercised muscle of normal subjects by creatine supplementation. Clin Sci (Lond). 1992;83: 367–374. 1327657 10.1042/cs0830367

[23] Gazzaneo MC, Orellana RA, Suryawan A, Tuckow AP, Kimball SR, Wilson FA, et al Differential regulation of protein synthesis and mTOR signaling in skeletal muscle and visceral tissues of neonatal pigs after a meal. Pediatr Res. 2011;70: 253–260. 10.1038/pr.2011.478 21654549 PMC3152601

[24] Vester B, Rasmussen K. High performance liquid chromatography method for rapid and accurate determination of homocysteine in plasma and serum. Eur J Clin Chem Clin Biochem. 1991;29: 549–554. 1760484 10.1515/cclm.1991.29.9.549

[25] Buchberger W, Ferdig M. Improved high-performance liquid chromatographic determination of guanidino compounds by precolumn derivatization with ninhydrin and fluorescence detection. J Sep Sci. 2004;27: 1309–1312. 15587280 10.1002/jssc.200401866

[26] Ratnam S, Wijekoon EP, Hall B, Garrow TA, Brosnan ME, Brosnan JT. Effects of diabetes and insulin on betaine-homocysteine S-methyltransferase expression in rat liver. Am J Physiol Endocrinol Metab. 2006;290: 933–939. 10.1152/ajpendo.00498.2005 16352668

[27] Bidlingmeyer BA, Cohen SA, Tarvin TL. Rapid analysis of amino acids using pre-column derivatization. J Chromatogr. 1984;336: 93–104. 6396315 10.1016/s0378-4347(00)85133-6

[28] Lamarre SG, Edison EE, Wijekoon EP, Brosnan ME, Brosnan JT. Suckling rat pups accumulate creatine primarily via de novo synthesis rather than from dam milk. J Nutr. 2012;140: 1570–1573. 10.3945/jn.110.125476 20660282

[29] Folch J, Lees M, Sloane Stanley GH. A simple method for the isolation and purification of total lipides from animal tissues. J Biol Chem. 1957;226: 497–509. 13428781

[30] Bartlett GR. Phosphorus assay in column chromatography. J Biol Chem. 1959;234: 466–468. 13641241

[31] Greenhaff PL, Bodin K, Soderlund K, Hultman E. Effect of oral creatine supplementation on skeletal muscle phosphocreatine resynthesis. Am J Physiol. 1994;266: 725–730. 10.1152/ajpendo.1994.266.5.E725 8203511

[32] Horn M, Frantz S, Remkes H, Laser A, Urban B, Mettenleiter A, et al Effects of chronic dietary creatine feeding on cardiac energy metabolism and on creatine content in heart, skeletal muscle, brain, liver and kidney. J Mol Cell Cardiol. 1998;30: 277–284. 9515004 10.1006/jmcc.1997.0590

[33] Syrotuik DG, Bell GJ. Acute creatine monohydrate supplementation: a descriptive physiological profile of responders vs. nonresponders. J Strength Cond Res. 2004;18: 610–617. 15320650 10.1519/12392.1

[34] Mora L, Sentandreu MÁ, Toldrá F. Contents of creatine, creatinine and carnosine in porcine muscles of different metabolic types. Meat Sci. 2008;79: 709–715. 10.1016/j.meatsci.2007.11.002 22063033

[35] Brault JJ, Terjung RL. Creatine uptake and creatine transporter expression among rat skeletal muscle fiber types. Am J Physiol Cell Physiol. 2003;284: 1481–1489. 10.1152/ajpcell.00484.2002 12570986

[36] Green AL, Hultman E, Macdonald IA, Sewell DA, Greenhaff PL. Carbohydrate ingestion augments skeletal muscle creatine accumulation during creatine supplementation in humans. Am J Physiol. 1996;271: 821–826. 10.1152/ajpendo.1996.271.5.E821 8944667

[37] Steenge G, Verhoef P. The effect of creatine and resistance training on plasma homocysteine concentration in healthy volunteers. Arch Intern Med. 2001;161: 1455–1456. 11386896 10.1001/archinte.161.11.1455

[38] Loike JD, Zalutsky DL, Kaback E, Mir AF, Miranda AF, Silverstein SC. Extracellular creatine regulates creatine transport in rat and human muscle cells. Proc Natl Acad Sci USA. 1998;85: 807–811. 10.1073/pnas.85.3.807 PMC279644 3422462

[39] Tachikawa M, Ikeda S, Fujinawa J, Hirose S, Akanuma S, Hosoya K. γ-Aminobutyric acid transporter 2 mediates the hepatic uptake of guanidinoacetate, the creatine biosynthetic precursor, in rats. Plos ONE. 2012;7: e32557 10.1371/journal.pone.0032557 22384273 PMC3288109

[40] Brosnan JT, Wijekoon EP, Warford-Woolgar L, Trottier NL, Brosnan ME, Bertolo RF. Creatine synthesis is a major metabolic process in neonatal piglets and has important implications for amino acid metabolism and methyl balance. J Nutr. 2009;139: 1292–1297. 10.3945/jn.109.105411 19474158

[41] da Silva RP, Nissim I, Brosnan ME, Brosnan JT. Creatine synthesis: hepatic metabolism of guanidinoacetate and creatine in the rat in vitro and in vivo. Am J Physiol Endocrinol Metab. 2009;296: 256–261. 10.1152/ajpendo.90547.2008 PMC2645018 19017728

[42] Edison EE, Brosnan ME, Meyer C, Brosnan JT. Creatine synthesis: production of guanidinoacetate by the rat and human kidney in vivo. Am J Physiol Renal Physiol. 2007;293: F1799–1804. 17928413 10.1152/ajprenal.00356.2007

[43] Harris RC, Lowe JA, Warnes K, Orme CE. The concentration of creatine in meat, offal and commercial dog food. Res Vet Sci. 1997;62: 58–62. 9160426 10.1016/s0034-5288(97)90181-8

[44] Hoffman DR, Cornatzer WE, Duerre JA. Relationship between tissue levels of S-adenosylmethionine, S-adenosylhomocysteine, and transmethylation reactions. Can J Biochem. 1979;57: 56–65. 427630 10.1139/o79-007

[45] Ostojic SM, Niess B, Stojanovic M, Obrenovic M. Creatine metabolism and safety profiles after six-week oral guanidinoacetic acid administration in healthy humans. Int J Med Sci. 2013;10: 141–147. 10.7150/ijms.5125 23329885 PMC3547211

[46] Li Z, Vance DE. Phosphatidylcholine and choline homeostasis. J Lipid Res. 2008;49: 1187–1194. 10.1194/jlr.R700019-JLR200 18204095

[47] Noga AA, Zhao Y, Vance DE. An unexpected requirement for phosphatidylethanolamine N-methyltransferase in the secretion of very low density lipoproteins. J Biol Chem. 2002;277: 42358–42365. 12193594 10.1074/jbc.M204542200

